# After Pulp Removal

**Published:** 1904-09

**Authors:** Chas. E. Parkhurst


After Pulp Removal.—That annoying particle of sensitive tissue at the apex of the root can ordinarily be removed by pumping carbolic acid and chloroform into the root with a smooth broach.—*Chas. E. Parkhurst, International Dental Journal.*

